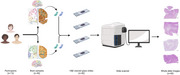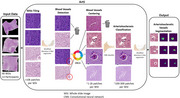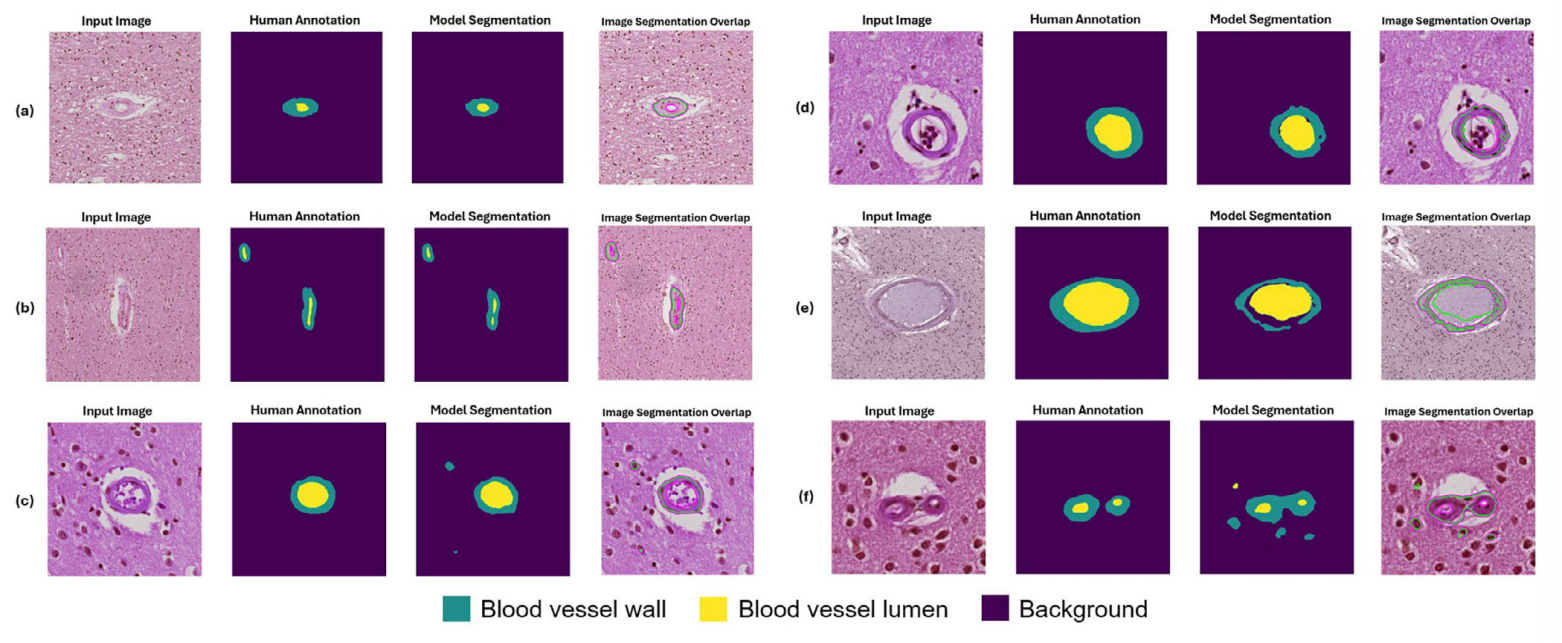# Automating quantitative morphometric analysis of arteriolosclerotic blood vessels using machine learning

**DOI:** 10.1002/alz70855_104156

**Published:** 2025-12-23

**Authors:** Jerry Jierui Lou, Peter Chang, Kiana D. Nava, Chanon Chantaduly, Hsin‐Pei Wang, William H Yong, Viharkumar Patel, Ajinkya J Chaudhari, Edwin Monuki, Elizabeth Head, Harry V. Vinters, Shino Magaki, Danielle J. Harvey, Chen‐Nee Chuah, Charles Decarli, Michael Keiser, Brittany N Dugger

**Affiliations:** ^1^ University of California, Irvine, School of Medicine, Irvine, CA, USA; ^2^ University of California, Davis, School of Medicine, Sacramento, CA, USA; ^3^ University of California, Irvine, Irvine, CA, USA; ^4^ University of California, Davis, Sacramento, CA, USA; ^5^ Geffen School of Medicine at UCLA, Los Angeles, CA, USA; ^6^ UCLA Easton Center for Alzheimer's Research, Los Angeles, CA, USA; ^7^ University of California, Los Angeles, Los Angeles, CA, USA; ^8^ University of California, Davis, Davis, CA, USA; ^9^ University of California, Davis School of Medicine, Sacramento, CA, USA; ^10^ Department of Neurology & Imaging of Dementia and Aging Laboratory, University of California Davis, Sacramento, CA, USA; ^11^ Alzheimer's Disease Research Center, University of California Davis, Sacramento, CA, USA; ^12^ University of California San Francisco, San Francisco, CA, USA

## Abstract

**Background:**

Current methods for assessment of brain arteriolosclerosis involve semi‐quantitative scales completed through manual histological examination of glass slides by humans. Such manual evaluations offer modest inter‐rater reliability at best and do not provide specific quantitative metrics. Not infrequently, arteriolosclerosis is assessed globally, as region‐specific evaluations with greater spatial resolution may be labor‐intensive and impractical for manual analysis.

**Method:**

To fill these gaps, we present a machine learning (ML)‐based algorithm – Arteriolosclerosis Segmentation (ArtS) followed by Sclerotic Index and Thickness Extractor (SITE) – that can automatically conduct morphometric analysis of arteriolosclerotic blood vessels on whole slide images (WSIs). We digitized hematoxylin and eosin‐stained glass slides of frontal and occipital lobe gray and/or white matter, collected from three brain banks (UCD ADRC, UCI ADRC, and UCLA ADRC), from 13 participants (total of 40 WSIs). The data was used to train three ML models within ArtS that, respectively, detect blood vessels, classify arteriolosclerosis, and segment arteriolosclerotic blood vessel walls and lumens.

**Result:**

For blood vessel detection, ArtS achieved a receiver operating characteristic curve (AUC‐ROC) of 0.79, 0.56 Dice score, and 2.53 Hausdorff distance on internal hold‐out testing, and a 0.77 AUC‐ROC, 0.74 Dice score, and 2.15 Hausdorff distance on external testing. For arteriolosclerosis classification, ArtS achieved a mean 0.94 accuracy and mean 0.69 AU‐ROC on 3‐fold cross validation; 0.86 accuracy and 0.87 AUC‐ROC on internal hold‐out testing; and a 0.77 accuracy and 0.83 AUC‐ROC on external testing. For arteriolosclerotic vessel segmentation, ArtS achieved a mean 0.68 Dice score, mean 7.63 Hausdorff distance, and mean 0.90 AUC‐ROC for 3‐fold cross validation; a 0.73 Dice score, 6.93 Hausdorff distance, and 0.92 AUC‐ROC for internal hold‐out testing; and a 0.71 Dice score, 7.80 Hausdorff distance, and 0.87 AUC‐ROC for external testing. For arteriolosclerotic blood vessels segmented by ArtS, SITE successfully calculated sclerotic indices and vessel wall thicknesses comparable to an expert.

**Conclusion:**

ArtS and SITE show promising potential for further optimization to create an assistive tool that enhances the current human‐based semi‐quantitative methods for assessment of vascular pathology.